# Acute Pericarditis: Descriptive Study and Etiology Determination in a Predominantly African American Population

**DOI:** 10.7759/cureus.1431

**Published:** 2017-07-06

**Authors:** Ahmad Awan, Fasil Tiruneh, Priscilla Wessly, Akbar Khan, Hasan Iftikhar, Sydney Barned, Daniel Larbi

**Affiliations:** 1 Department of Internal Medicine, Howard University Hospital; 2 Health Information Management, Howard University Hospital

**Keywords:** pericardial disease, pericarditis, african-american

## Abstract

INTRODUCTION

­­­Acute pericarditis is the most commonly encountered manifestation of pericardial disease (incidence: 0.2 percent to 0.5 percent in hospitalized patients). However, data regarding manifestations, workup, and the management of acute pericarditis in the African American population is lacking. This study aims to collect and analyze more clinical data related to acute pericarditis in this understudied population.

METHODOLOGY

We conducted a retrospective chart review of all patients managed for acute pericarditis at a university hospital serving a predominantly African American population. A total of 78 charts were reviewed during the period of study from January 2011 to July 2016. Out of these, nine charts were excluded due to poor data. We descriptively analyzed data regarding presenting symptoms, underlying etiologies, co-morbidities, investigation results, management strategies, and prognoses.

RESULTS

We found an equal number of males and females in our study population. The most common comorbid conditions were hypertension, chronic kidney disease, and diabetes mellitus (in order of incidence). The most common presentation of symptomatic pericarditis consisted of chest pain, dyspnea, tachycardia, and tachypnea. Electrocardiogram (EKG) findings included diffuse ST elevation (15 percent) and sinus tachycardia (41 percent). Leukocytosis was seen in 15 percent of the patients. The most common etiology noted in our patient population was idiopathic and was treated with NSAIDS.

CONCLUSION

As compared to other populations, the incidence of uremic pericarditis and pericarditis secondary to cardiac etiologies is slightly higher in the African American population; however, the clinical presentation, examination and laboratory findings, as well as investigations, are remarkably similar.

## Introduction

Acute pericarditis is a common disorder in several clinical settings, where it may be the first manifestation of a systemic disease or may represent an isolated process. Though epidemiologic studies are limited, the incidence of this disorder is recorded to be between 0.1 and 0.2 percent of hospitalized patients, and five percent of the patients admitted to the emergency department with non-ischemic chest pain. Despite the fact that this is a common disease with high prevalence and is highly researched, data regarding prevalence, manifestations, workups, and management in the African American population are lacking. This study aims to collect and analyze more clinical data related to acute pericarditis in this understudied population.

## Materials and methods

We conducted a retrospective descriptive chart review of all patients who were treated at Howard University Hospital, Washington, D.C., with a diagnosis of acute pericarditis based on the international classification of diseases, ninth revision code (ICD-9), during the time period of January 2011 to July 2016. We descriptively analyzed clinical presentation, physical examination findings, lab investigations, management, underlying etiologies, and mortality data in our study population. The data was recorded as numbers and analyzed as percentages of cases. The recorded comorbid conditions in the study population included hypertension, diabetes mellitus, hyperlipidemia, chronic kidney disease, coronary artery disease, congestive heart failure, and autoimmune and liver diseases. Special attention was given to electrocardiogram (EKG) findings. The EKG findings were divided into normal EKG, sinus tachycardia, nonspecific ST-T wave pattern, and diffuse ST elevation. Similarly, we also recorded the number of patients who had a transthoracic echocardiogram (TTE) done. In patients who had an echocardiogram completed, findings were classified into normal echocardiogram, pericardial effusion, and regional or global wall motion abnormality. The treatment administered was categorized into non-steroidal anti-inflammatory agents (NSAIDs) alone or a NSAID in combination with colchicine, colchicine alone, hemodialysis, and pericardiocentensis with or without drain placement. The causes of pericarditis were divided into infectious causes, cardiac causes, autoimmune diseases, traumatic causes, and idiopathic and malignant etiologies.

## Results

We identified 69 patients who were diagnosed with acute pericarditis based on ICD-9. Out of all of the patient charts, nine were discarded due to poor or incomplete documentation. Of the remaining 60 identified patients, 33 (55 percent) were male and 27 (33 percent) were female (Table 1). The majority of our patients were the African American population (94 percent), followed by the Hispanic population (5 percent), and the Asian population (1 percent). Hypertension was the most common comorbid condition at 46 percent, followed by chronic kidney disease at 28 percent. About 17 percent had a history of congestive heart failure. Comorbid conditions are tabulated in Table 2. The most common presenting symptom was chest pain (73 percent) followed by cough (62 percent), and dysphagia (25 percent). The most common physical examination finding was tachycardia (65 percent) followed by tachypnea (46.7 percent). Pericardial friction rub was documented in 30 percent of the patients who had this disorder. Findings in history and physical examinations are tabulated in Table 3.

**Table 1:** Gender Distribution of Study Population

	Male N (%)	Female N (%)	Total N
African Americans	31 (51.7)	25 (41.7)	56
Hispanics	2 (3.3)	1 (1.6)	3
Asian	0 (0)	1 (1.6)	1
Total	33 (55)	27 (45)	60

**Table 2:** Comorbid Condition in Study Population HTN: Hypertension, DM: Diabetes Mellitus, HLD: Hyperlipidemia, CKD: Chronic Kidney Disease, CAD: Coronary Kidney Disease, CHF: Congestive Heart Failure

CONDITION	N (%)
HTN	28 (46.7)
DM	12 (20)
HLD	11 (18.3)
CKD	17 (28.3)
CAD	6 (10)
CHF	10 (16.7)
Auto-immune	4 (6.7)
Liver Diseases	3 (5)

 

**Table 3:** Symptoms and Physical Examination Findings in Patients with Pericarditis

Symptoms and Physical Exam Findings	Total N (%)
Chest pain	44 (73.3)
Cough	37 (61.6)
Dysphagia	15 (25)
Tachycardia	39 (65)
Tachypnea	28 (46.7)
Pericardial friction rub	18 (30)

Almost 15 percent of our patients had a normal EKG. Sinus tachycardia was the most common EKG abnormality, seen in 41.6 percent of our patients, followed by nonspecific ST-T wave abnormalities, which were seen in 16.6 percent of the patient population. The characteristic diffuse ST segment elevation with PR depression was seen only in 15 percent of the cases.

Our findings show that TTE was not done in 17 percent of the study population. In the remaining 83 percent, it was found normal in only 17 percent of the patients. Pericardial effusion was the most common echocardiographic abnormality seen in 60 percent of the patients. Twenty-four percent of the patients had findings suggestive of tamponade. Echocardiographic findings are reported in Table 4.

**Table 4:** Echocardiographic Findings

Echo Finding	Total N (%)
Normal	10 (16.7)
Pericardial effusion	36 (60)
Wall motion abnormality	4 (6.7)
Pericardial tamponade	14 (23.3)
Echo not done	10 (16.7)

Fifty percent of the patients had leukocytosis and elevation in creatinine from the baseline. The C-Reactive Protein (CRP) test was not done in approximately 72 percent of the patients. Yet, in 28 percent of the cases in which it was done, 20 percent of the patients had elevation in CRP. Lab findings are documented in Table 5.

**Table 5:** Laboratory Findings CRP: C-Reactive Protein, HIV: Human Immunodeficiency Virus infection, ANA: Antinuclear antibody

Labs	Elevated N (%)	Normal N (%)	Not Done N (%)
Leukocytosis	30 (50)	30 (50)	0 (0)
CRP	12 (20)	5 (8.3)	43 (71.7)
HIV	10 (16.7)	32 (53.3)	18 (30)
ANA	2 (3.3)	17 (28.3)	41 (68.3)
Creatinine	29 (48.3)	31 (51.7)	0 (0)

The most common cause of acute pericarditis was idiopathic (33 percent) followed by infectious etiology (25 percent) and uremic pericarditis (13.3 percent). Cardiac diseases and malignancy were identified in 10 percent of the cases. There was less than one percent mortality in our study population. These findings are reported in Table 6.

**Table 6:** Etiology of Pericarditis

Etiology	Total N (%)
Idiopathic	20 (33.3)
Infection	15 (25)
Uremic	8 (13.3)
Cardiac	6 (10)
Malignancy	6 (10)
Traumatic	3 (5)
Autoimmune	2 (3.3)
Total	60

Most patients were treated with either nonsteroidal anti-inflammatory drugs (NSAIDs) alone or in combination with Colchicine. Hemodialysis was done in 13.3 percent of the cases that had uremic pericarditis. Pericardiocentesis was done in 23 percent of the cases and almost exclusively in patients who had pericardial tamponade.

## Discussion

Acute pericarditis is a relatively common heart condition seen in several clinical settings, where it may be the first manifestation of a systemic disease or may represent an isolated process. Though epidemiologic studies are lacking, acute pericarditis is recorded in 0.1 to 0.2 percent of hospitalized patients and in five percent of the patients admitted to the emergency department with non-ischemic chest pain [1]. 

Acute pericarditis can present itself in a variety of ways, depending on the underlying etiology. Chest pain is the most common clinical symptom. Chest pain is typically described as "pleuritic," worsened with inspiration and deep breathing, and is presented in the anterior part of the chest. According to some studies, chest pain is present in more than 95 percent of the patients [2]. In our study, chest pain was present in more than 70 percent of the patients and was the most common abnormality. Coughing is another recognized symptom of this disorder. More than 60 percent of the patients had a cough. Dysphagia was rarely reported in clinical settings as a presenting complaint of acute pericarditis. Our study findings showed almost 25 percent of patients presented with dysphagia.

The presence of pericardial friction rub is highly specific for acute pericarditis. The classic pericardial friction rub consists of three phases, corresponding to the movement of the heart during atrial systole, ventricular systole, and the rapid early filling of early diastole [3]. Studies indicate that it is present in almost 35 percent of patients with pericarditis [4]. In our study, the prevalence of acute pericarditis was found to be 30 percent. 

CRP is a relatively inexpensive, yet sensitive, test for the diagnosis of this disorder; and it is relatively underutilized. Studies have shown that it is usually elevated in almost 75 percent of the cases [5]. In our study, we found that CRP was an underutilized marker in its diagnosis. It is not completed or ordered in almost 72 percent of the cases. However, when it is done, it was positive in more than 70 percent of the cases.

Typically, in patients with acute pericarditis, EKG progresses through four stages:

Stage I: Diffuse ST elevation with reciprocal ST depression in I and aVL

Stage II: Normalization of ST and PR segments

Stage III: Development of diffuse T-wave inversions

Stage IV: Normalization of ECG or indefinite persistence of T-wave inversions

In our study, diffuse ST-T wave elevation was present in only 15 percent of the patients. Our most common EKG findings were sinus tachycardia, a nonspecific finding. On the basis of our study, we concluded that although diffuse ST elevation is characteristic of acute pericarditis, its absence does not rule out the diagnosis. We recommend relying on history and physical exam findings.

The presence of pericardial effusion is one aspect of diagnostic criteria [6]. Studies have shown that pericardial effusions are typically present in almost 60 percent of patients with pericardial effusion, and tamponade is present in only 5 percent of cases with pericardial effusion [2]. Like other studies, our study also showed that pericardial effusion was present in almost 60 percent of the cases; conversely, our study showed a higher incidence of pericardial tamponade (23 percent). Based on our research, we recommend that all patients with a suspected or an established diagnosis of acute pericarditis should be receiving a transthoracic echocardiogram to rule out life-threatening pericardial tamponade.

The yield of diagnostic evaluation to determine the etiology of acute pericarditis is low. Most studies have shown that specific underlying etiology is found in only 17 percent of the cases and more than 80 percent of the cases are secondary to infectious etiology or unknown causes [7-9]. The most common established diagnoses are neoplasia (five percent), tuberculosis (four percent), and autoimmune causes (three percent). In our study, idiopathic and infectious etiologies together contributed towards 58 percent of the cases. Despite this, surprisingly, the incidence of uremic pericarditis was high in our population (13 percent) compared to other populations (less than 3 percent) [9]. This could be secondary to a high incidence of chronic kidney disease in our population; however, the exact reason cannot be determined without further studies. Similarly, the incidence of pericarditis secondary to cardiac etiologies was remarkably high in our study population (10 percent). The exact mechanism behind this high incidence is also unknown.

Our study had several limitations. We had a small sample size, given our population was culled from a single urban hospital in Washington, D.C. The focus on African American patients also means our findings may not generally be applied to other population subgroups. 

## Conclusions

As compared to other populations, the incidence of uremic pericarditis and pericarditis secondary to cardiac etiologies is slightly higher in the African American population; however, the clinical presentation, examination and laboratory findings, as well as investigations, are remarkably similar. Further studies are needed to validate these findings and identify the confounding factors, such as age, comorbid conditions, gender differences, and socioeconomic conditions.
